# Blueberry Polyphenols Increase Nitric Oxide and Attenuate Angiotensin II-Induced Oxidative Stress and Inflammatory Signaling in Human Aortic Endothelial Cells

**DOI:** 10.3390/antiox11040616

**Published:** 2022-03-23

**Authors:** Rami S. Najjar, Shengyu Mu, Rafaela G. Feresin

**Affiliations:** 1 Department of Nutrition, Georgia State University, Atlanta, GA 30302, USA; rnajjar1@student.gsu.edu; 2 Department of Pharmacology & Toxicology, University of Arkansas for Medical Sciences, Little Rock, AR 72205, USA; smu@uams.edu; 3 Department of Nutrition & Dietetics, University of Arkansas for Medical Sciences, Little Rock, AR 72205, USA

**Keywords:** NRF2, antioxidant enzymes, redox signaling, reactive oxygen species, NADPH oxidases, endothelial dysfunction, hypertension, cardiovascular

## Abstract

Accumulating evidence indicate that blueberries have anti-hypertensive properties, which may be mainly due to its rich polyphenol content and their high antioxidant capacity. Thus, we aimed to investigate the mechanisms by which blueberry polyphenols exert these effects. Human aortic endothelial cells (HAECs) were incubated with 200 µg/mL blueberry polyphenol extract (BPE) for 1 h prior to a 12 h treatment with angiotensin (Ang) II, a potent vasoconstrictor. Our results indicate that Ang II increased levels of superoxide anions and decreased NO levels in HAECs. These effects were attenuated by pre-treatment with BPE. Ang II increased the expression of the pro-oxidant enzyme NOX1, which was not attenuated by BPE. Pre-treatment with BPE attenuated the Ang II-induced increase in the phosphorylation of the redox-sensitive MAPK kinases, SAPK/JNK and p38. BPE increased the expression of the redox-transcription factor NRF2 as well as detoxifying and antioxidant enzymes it transcribes including HO-1, NQO1, and SOD1. We also show that BPE attenuates the Ang II-induced phosphorylation of the NF-κB p65 subunit. Further, we show that inhibition of NRF2 leads to a decrease in the expression of HO-1 and increased phosphorylation of the NF-κB p65 subunit in HAECs treated with BPE and Ang II. These findings indicate that BPE acts through a NRF2-dependent mechanism to reduce oxidative stress and increase NO levels in Ang II-treated HAECs.

## 1. Introduction

Oxidative stress and inflammation are major drivers of cellular and molecular events that can lead to the development of hypertension, a multifactorial and multiorgan disease [1] that affects 49.6% adults in the United States [2]. Oxidative stress is characterized by accumulation of reactive oxygen species (ROS), which is caused by “an imbalance between oxidants and antioxidants in favor of oxidants” [3]. ROS is a major signaling molecule mediating the actions of angiotensin (Ang) II, a vasoactive peptide that can lead to vascular oxidative stress and inflammation, vasoconstriction, tissue hypertrophy and fibrosis [4,5,6,7]. Through its binding to Ang II type 1 receptor (AT_1_R), a G protein-coupled receptor, Ang II can lead to activation of NADPH oxidases (NOX) resulting in increased production of ROS such as hydrogen peroxide (H_2_O_2_) and superoxide anion (O_2_^•−^). Superoxide anion reacts with the vasodilator nitric oxide (NO) forming peroxynitrite (OONO^−^), thereby reducing NO bioavailability [8,9]. Additionally, increased ROS causes endothelial NO synthase (eNOS) uncoupling favoring production of O_2_^•−^ over NO, which further increases ROS in endothelial cells [10]. Altogether, these events can lead to endothelial dysfunction [11] and high blood pressure [12].

Ang II-AT_1_R binding also promotes the phosphorylation and activation of the redox-sensitive threonine/serine kinases such as protein kinase B (Akt) and mitogen activated protein kinases (MAPKs): p38MAPK, stress-activated protein kinase (SAPK)/Jun amino-terminal kinases (JNK), and extracellular signal-regulated kinase (ERK)1/2, which play key roles in cell differentiation, proliferation, migration, hypertrophy, and fibrosis [13,14,15]. In fact, administration of p38MAPK inhibitor reduced blood pressure and cardiac hypertrophy in Ang II-infused male rats [16]. Further, Ang II-AT_1_R binding activates the pro-inflammatory transcription factor nuclear factor kappa-light-chain-enhancer of activated B cells (NF-κB), which is mediated by NOX-derived ROS production and MAPK activation [14] and results in up-regulation of pro-inflammatory genes, cytokine production, and tissue fibrosis. Zhao et al. [17] demonstrated that NOX4 transgenic mice infused with Ang II had increased oxidative stress, cardiac fibrosis, and hypertrophy and that these effects were mediated by Akt-NF-κB activation. 

Nuclear factor erythroid 2-related factor (NRF) 2 is a transcription factor found sequestered in the cytoplasm by Kelch-like ECH-associated protein (KEAP) 1 [18]. In the presence of oxidative stress, NRF2 dissociates from KEAP1 and translocates to the nucleus where it binds to antioxidant response element (ARE) inducing the transcription of several detoxifying and antioxidant enzymes including heme oxygenase (HO)-1, NADH dehydrogenase (quinone) (NQO) 1 [18], and partially mediates superoxide dismutase (SOD), catalase (CAT), and glutathione peroxidase (GPx) 1 [19,20]. SOD is a major antioxidant enzyme responsible for converting O_2_^•−^ into H_2_O_2_ while peroxidases such as CAT and GPx1 convert H_2_O_2_ into water and oxygen. Increased expression of NRF2 and the enzymes it transcribes can potentially increase NO production and bioavailability promoting endothelium-dependent vasodilation and lowering blood pressure [21]. 

Counteracting ROS, in particular O_2_^•−^, production and/or accumulation is an attractive approach to improving endothelial function. Blueberries are native to North America and widely consumed in the United States. They are rich in flavonoids especially anthocyanins, including cyanidin 3-O-galactoside, delphinidin 3-O-galactoside, and malvidin 3-O-galactoside, as well as the flavonol myricetin, which is unique among other berries [22]. Blueberries have strong antioxidant capacity and accumulating evidence supports their antihypertensive properties. For instance, consumption of wild blueberries improved endothelial function in a dose-dependent manner and reduced NOX activity in neutrophils, which was closely tied to serum fluctuation in blueberry-derived polyphenols in healthy men [23]. Further, we showed that daily consumption of blueberries for eight weeks reduced blood pressure and increased levels of plasma NO in hypertensive postmenopausal women [24]. Similarly, Basu et al. [25] observed a reduction of blood pressure in adults with metabolic syndrome who consumed blueberry for eight weeks. Other groups reported that blueberry consumption for six weeks [26] and six months [27] improved endothelial function in subjects with metabolic syndrome. Interestingly, Curtis et al. [28] showed that blueberries were ineffective at improving endothelial function post-prandially in adults with metabolic syndrome. This may be due to the method by which blueberries were provided, as they were part of a milk-based smoothie, and milk-derived proteins tend to neutralize the antioxidant effects of blueberries and inhibits absorption of polyphenols [29]. Nonetheless, further evidence from animal studies indicate that blueberries reduce blood pressure and decrease renal oxidative stress in spontaneously hypertensive rats and spontaneously hypertensive stroke-prone rats [30,31]. Thus, the aim of this study was to gain insight into the mechanisms by which blueberry polyphenols exert their antihypertensive effects using human aortic endothelial cells (HAECs) treated with Ang II.

## 2. Materials and Methods

### 2.1. Chemicals and Reagents

Ang II (Bachem); chloromethyl 2′,7′-dichlorodihydrofluorescein diacetate (H_2_DCFDA), Gallic acid (Thermo Fischer Scientific, Waltham, MA, USA); Folin–Ciocalteu reagent, 4,5-diaminofluorescein diacetate (DAF-2 DA), TOX8, radioimmunoprecipitation assay (RIPA) buffer, phosphatase inhibitor cocktail 1 and 2 and protease inhibitor cocktail (Sigma-Aldrich, St. Louis, MO, USA); EC Basal Growth Medium MV, EC Growth Medium MV Supplement Mix (Promo Cell, Heidelberg, Germany); Dihydroethidium (DHE, Thermo Fisher Scientific, Rockford, IL, USA); rabbit polyclonal antibodies against: p-p38MAPK (cat#: 4511), p38MAPK (cat#: 8690), p-SAPK/JNK (cat#: 4668), SAPK/JNK (cat#:9 252), p-ERK1/2 (cat#: 9101), ERK1/2 (cat#:9102), p-NF-κB p65 (Cat#: 3033), NF-κB p65 (cat#: 4764), p-Akt (cat#: 4060) GPx1 (cat#: 3206), catalase (cat#: 14097), SOD1 (cat#: 2770), SOD2 (cat#: 13141), and HO-1 (cat#: 5061) (Cell Signaling, Danvers, MA, USA); rabbit polyclonal antibodies against: NOX2 (cat#: ab180642), NOX4 (ab133303) and NOX5 (cat#: ab191010) (Abcam, Cambridge, UK); rabbit polyclonal antibodies against: NRF2 (cat#: NBP1-32822) and NOX1 (cat#: NBP1-31546) (Novus Biologicals, Centennial, CO, USA); sheep polyclonal antibodies against: NQO1 (cat#: AF7567) (R&D systems, Minneapolis, MN, USA); and mouse monoclonal antibodies against: Akt (cat#: 2920) and β-actin (cat#: 3700) (Cell Signaling, Danvers, MA, USA).

### 2.2. Extraction of Blueberry Polyphenols 

Polyphenol extraction and purification of Highbush freeze-dried blueberry powder, derived from a 50/50 blend of Tifblue (*Vaccinium virgatum*) and Rubel (*Vaccinium corymbosum*), was performed as described by Feresin et al. [32]. Briefly, freeze-dried blueberry powder was extracted with 80% ethanol in an ultrasonic bath under subdued light with nitrogen purging to avoid oxidation. Solution was filtered, evaporated, and freeze-dried prior removal of organic molecules using chloroform. The aqueous fraction was collected and combined with ethyl acetate before being evaporated and freeze-dried again. Samples were stored at −20 °C for later analysis and use.

### 2.3. Total Polyphenol Content

The total polyphenol content of BPE was evaluated colorimetrically using Folin–Ciocalteu reagent according to a previously published protocol with some modifications [33]. BPE was dissolved in 90% EtOH at a concentration of 1 mg/mL. Gallic acid was used as a standard and dissolved in 90% EtOH at a concentration range of 0–800 μM. BPE and gallic acid standards were pipetted in triplicates into a clear, 96-well plate (20 μL/well), and 40 μL of 10% Folin–Ciocalteu reagent was added to each well containing sample and standard, followed by brief shaking. Then, 140 μL of Na_2_CO_3_ (700 mM) was added to these wells, followed by a 10-min incubation at room temperature. The absorbance was then read at 765 nm in a plate reader (Synergy HT, Biotek, Winooski, VT, USA). 

### 2.4. Total Anthocyanin Content 

The total anthocyanin content of BPE was evaluated colorimetrically using the pH-differential method [34]. A solution of KCl (25 mM, pH 1.0) and CH_3_CO_2_Na·3H_2_O (400 mM, pH 4.5) was created in water and BPE was dissolved in each at 1 mg/mL. The absorbance of both BPE solutions was measured at 520 nm and 700 nm in a clear, 96-well plate, and cyanidin-3-glucoside equivalents were calculated with the following equation:

$$\mathrm{Anthocyanin}\;\mathrm{pigment}\;\left(\mathrm{cyanidin}-3-\mathrm{glucoside}\;\mathrm{equivalents}\frac{\mathrm{mg}}{L}\right)=\frac{A*\mathrm{MW}*\mathrm{DF}*10^{3}}{\varepsilon *1}$$

where

- A = (A520–A700 nm) pH1.0 − (A520–A700 nm) pH4.5.
- MW (molecular weight) = 449.2 g/mol for cyanidin-3-glucoside.
- DF = dilution factor.
- 1 = pathlength in cm.
- ε = 26,900 molar extinction coefficient in L × mol^−1^ × cm^−1^ for cyanidin-3-glucoside.
- 10^3^ = factor for conversion from g to mg

### 2.5. BPE Total Antioxidant Capacity

The total antioxidant capacity was assessed using the ferric reducing antioxidant power (FRAP) assay [35]. A master mix was prepared containing 300 mM CH_3_COONa·3H_2_O, 10 mM TPTZ in 40 mM HCl, and 20 mM FeCl·6H_2_O at a ratio of 10:1:1. Fe^2+^SO_4_·7H_2_O was used as a standard ranging from 0 to 500 μM. BPE was dissolved in water at a concentration of 1 mg/mL. Then, 20 μL of BPE and standards were pipetted into a clear, 96-well plate with 180 μL of master mix. After 10 min of incubation at room temperature, absorbance was read at 593 nm.

### 2.6. Cell Culture

HAECs were cultured in flat-bottom flasks containing complete media (EC Basal Growth Medium MV supplemented with EC Growth Medium MV Supplement Mix and 1% antibiotics at 37 °C and 5% CO_2_. Media was changed every two days. When the cells reached approximately 80% confluency, they were detached using 0.04% trypsin/EDTA solution, then centrifuged and re-suspended in media. HAECs were seeded in complete media with a density of 5 × 10^3^ cells per cm^2^ in 60 mm dishes for protein expression and 96-well black plates for cell viability, ROS, and NO measurements. Upon reaching 70% confluency, cells were treated with blueberry polyphenol extract (BPE; 200 µg/mL) for 1 h, followed by stimulation with 200 nM of Ang II for 12 h in starvation medium containing 0.5% FBS (10% of supplement quantity used in basal medium).

### 2.7. Cell Viability

Cells were treated with a BPE concentration range of 200–1000 µg/mL for 24 h in starvation medium. Following treatment, medium was rinsed with warm PBS, and fresh starvation medium was added. TOX8 was added to each well at a concentration of 10% of volume of medium. After incubation for 2 h in 37 °C and 5% CO_2_, fluorescent intensity was read at Ex/Em of 530/590 in a microplate reader (Synergy HT, Biotek).

### 2.8. ROS Measurements

Methods for ROS detection were modified from a prior investigation [36]. Cells were washed with Dulbecco’s phosphate-buffered saline (DPBS) twice and incubated with 10 µM of DHE or H_2_DCFDA fluorescent probes for 30 min in 37 °C 5% CO_2_. Fluorescence was read using a microplate reader at 480/590 (Ex/Em) for DHE and 405/570 (Ex/Em) for H_2_DCFDA.

### 2.9. Intracellular NO

Methods were modified from Rathel et al. [37]. In brief, DAF-2 DA was added to each well at a final concentration of 5 µM. After a 30-min incubation in 37 °C and 5% CO_2_, cells were washed twice in warm PBS, then left in warm phenol red-free medium for imaging and quantitative analysis at a fluorescent intensity of 495 nm/515 nm (Ex/Em).

### 2.10. NOS Activity

Cells were collected according to instructions in a commercially available NOS assay kit (cat#: ab211083; Abcam, Waltham, MA, USA). NOS assay buffer (provided by the manufacturer) was supplemented with protease and phosphatase inhibitor cocktails then briefly sonicated at 20% amplitude for complete cell lysis. NOS activity was then assessed according to kit instructions.

### 2.11. Western Blot Analysis

As described elsewhere [38], cells were lysed in RIPA supplemented with protease and phosphatase inhibitor cocktails, followed by centrifugation at 20,000× *g* for 20 min for collection of supernatants. Protein concentration of lysates were determined using the DC protein assay kit (BioRad Laboratories, Hercules, CA, USA). Samples containing 30 µg of protein were mixed with 4× Laemli supplemented with 10% 2-mercaptoethanol (BioRad Laboratories, Hercules, CA, USA), placed in a dry bath at 70 °C for 10 min and separated using SDS-PAGE. Gels were transferred to polyvinylidene difluoride (PVDF) membranes (BioRad Laboratories, Hercules, CA, USA) using Trans-Blot Turbo (BioRad Laboratories, Hercules, CA, USA). The membrane was incubated with 5% non-fat dried milk (NFDM) in Tris-buffered saline (TBS; 20 mM Tris, pH 7.5, 130 mM NaCl) containing 0.5% Tween-20 (TBS-T) for 20 min on a shaker, then washed with TBS-T 3 × 5 min before incubating with the primary antibody solution (1:1000 dilution in TBS-T with 5% BSA and 0.1% sodium azide) overnight at 4 °C on a shaker. Next, the membranes were washed 3 × 5 min with TBS-T, then incubated with the secondary antibody solution containing 5% NFDM in TBS-T at a 1:10,000 dilution for 1 h at room temperature. The membranes were washed for an additional 3 × 5 min using TBS-T, then incubated in Immobilon Forte Western HRP Substrate (EMD Millpore, Billerica, MA, USA) for imaging using the ChemiDoc Imaging Systems (BioRad Laboratories, Hercules, CA, USA). Density of the protein bands was quantified using Image Lab 6.0 (BioRad Laboratories, Hercules, CA, USA).

### 2.12. Statistical Analysis

Data distribution was examined using Shapiro–Wilk. Data were found to be normally distributed thus one-way analysis of variance (ANOVA) was used next followed by Tukey–Kramer post hoc test for pairwise comparisons. Significant differences were determined at *p* ≤ 0.05. Values are presented as mean ± standard deviation of the mean (SD). Data analyses were performed using GraphPad Prism software (La Jolla, CA, USA).

## 3. Results

### 3.1. Polyphenol Content of BPE

The polyphenol and anthocyanin content as well as antioxidant capacity of ethanol-based BPE used in this study are described in Table 1. Based on previous cell culture studies with berry extracts conducted in our laboratory [36,38] and cell viability data (not shown), 200 µg/mL BPE was used to perform further experiments.

### 3.2. Effects of BPE on NO and ROS Levels in Ang II-Treated HAECs

**Figure 1:**
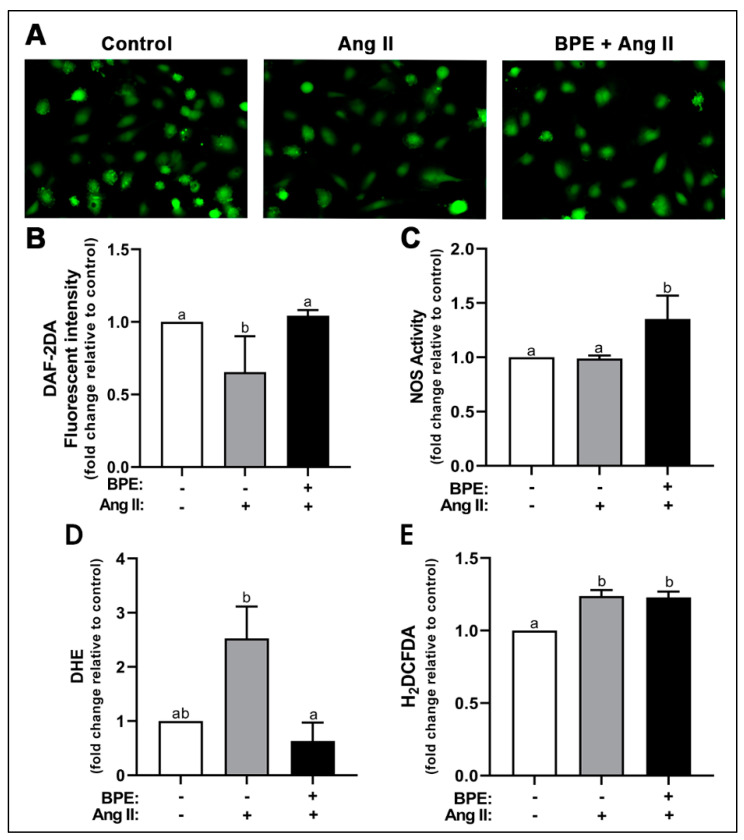
**Blueberry polyphenol extract (BPE) prevents the decrease in nitric oxide (NO) levels and the increase in superoxide production induced by angiotensin (Ang) II in human aortic endothelial cells (HAECs).** HAECs were treated with 200 µg/mL of BPE for 1 h then treated with 200 nM of Ang II for 12 h. NO levels were visualized (**A**) and quantified (**B**) after 30 min incubation with DAF-2DA. NO synthase (NOS) activity (**C**). ROS levels were determined after 30 min incubation with DHE (**D**) or H_2_DCFDA (**E**). Data are expressed as mean ± SD from three independent experiments. Values that do not share the same letter are significantly different from each other (*p* ≤ 0.05).

We first examined the effects of Ang II and BPE on NO levels. Ang II significantly decreased levels of NO (0.65 ± 0.25-fold; n = 3; *p* = 0.008) compared to control (Figure 1A,B). Pre-treatment with BPE significantly prevented that decrease (1.04 ± 0.04-fold; n = 3; *p* = 0.005) (Figure 1A,B). To test whether these effects were mediated by the actions of Ang II and BPE on the synthesis of NO, we measured NOS activity. Ang II had no effects on NOS activity in HAECs while BPE significantly increased NOS activity compared to Ang II (1.35 ± 0.22 vs. 0.99 ± 0.03-fold, respectively; n = 3; *p* = 0.03) and control (*p* = 0.03) (Figure 1C). This may indicate that the reduction in NO metabolite levels observed with Ang II is due to a decrease in NO bioavailability rather than a decrease in NO production. To test this hypothesis, we investigated the effects of Ang II and BPE on ROS levels using DHE and H_2_DCFDA. These are fluorescent probes that allow for the detection of O_2_^•−^ and H_2_O_2_ as well as hydroxyl and peroxyl radicals, respectively. Ang II increased levels of O_2_^•−^ (2.52 ± 1.02-fold; n = 3; *p* = 0.07) compared to control, which trended towards significance (Figure 1D). Pre-treatment with BPE significantly attenuated the increase in O_2_^•−^ induced by Ang II (0.63 ± 0.59-fold; n = 3; *p* = 0.03) (Figure 1D). Ang II induced a significant increase in H_2_O_2_ levels (1.24 ± 0.08-fold; n = 4; *p* = 0.002) compared to control; however, BPE did not prevent or attenuated this effect (1.23 ± 0.08-fold; n = 4; *p* = 0.98) (Figure 1E).

### 3.3. BPE Attenuated the Phosphorylation of SAPK/JNK and p38MAPK in Ang II-Treated HAECs

**Figure 2:**
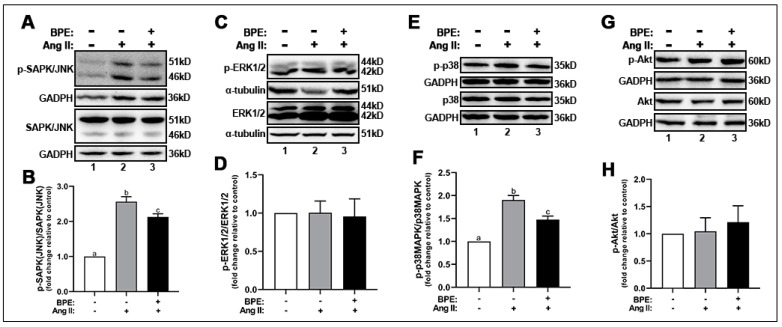
**Blueberry polyphenol extract (BPE) attenuates SAPK/JNK and p38 phosphorylation in angiotensin (Ang) II-treated human aortic endothelial cells (HAECs).** HAECs were treated with 200 µg/mL of BPE for 1 h then treated with 200 nM of Ang II for 12 h. Protein expression of phosphorylated and total SAPK/JNK (**A**,**B**), ERK1/2 (**C**,**D**), p38 (**E**,**F**), and Akt (**G**,**H**) were determined by Western blot. Quantification was performed using Image Lab (Bio-Rad Laboratories, Inc., Hercules, CA, USA). Data are expressed as mean ± SD from three (SAPK/JNK), four (Akt) and five (ERK1/2 and p38) independent experiments. Values that do not share the same letter are significantly different from each other (*p* ≤ 0.05).

Next, we explored the effects of Ang II and BPE on ROS signaling by measuring the expression of the phosphorylated redox-sensitive MAPKs and Akt. Ang II increased the phosphorylation of SAPK/JNK (2.56 ± 0.25-fold; n = 3; *p* ˂ 0.0001) (Figure 2A,B) and p38MAPK (1.9 ± 0.22-fold; n = 5; *p* = 0.002) (Figure 2E,F) compared to control. Exposure of HAECs to BPE prior to Ang II treatment significantly attenuated the SAPK/JNK (2.13 ± 0.16-fold; n = 3; *p* = 0.04) (Figure 2A,B) and p38MAPK (1.48 ± 0.17-fold; n = 5; *p* = 0.004) (Figure 2E,F) phosphorylation. Interestingly, expression of total and phosphorylated ERK1/2 (Figure 2C,D) and Akt (Figure 2G,H) was not affected by Ang II or BPE.

### 3.4. BPE Did Not Attenuate Ang II-Induced Increase in NOX1 Expression in HAECs

**Figure 3:**
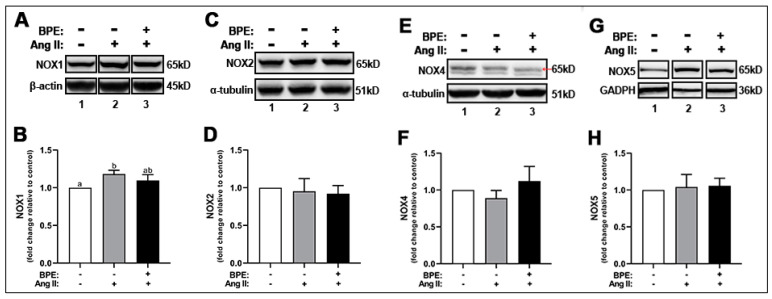
**Blueberry polyphenol extract (BPE) did not affect the expression of NADPH oxidases (NOX) in angiotensin (Ang) II-treated human aortic endothelial cells (HAECs).** HAECs were treated with 200 µg/mL of BPE for 1 h then treated with 200 nM of Ang II for 12 h. Protein expression of NOX1 (**A**,**B**), NOX2 (**C**,**D**), NOX4 (**E**,**F**), and NOX5 (**G**,**H**) were determined by Western blot. Quantification was performed using Image Lab (Bio-Rad Laboratories, Inc.). Data are expressed as mean ± SD from three (NOX1 and NOX4) and five (NOX2 and NOX5) independent experiments. Values that do not share the same letter are significantly different from each other (*p* ≤ 0.05).

As indicated above, Ang II led to increases in ROS levels. NOXs are one of the major ROS-producing enzymes in HAECs. Therefore, we hypothesized that this increase in ROS could be due to Ang II-induced increased expression of NOX enzymes in these cells. Ang II increased the expression of NOX1 (1.18 ± 0.05-fold; n = 3; *p* = 0.01) compared to control (Figure 3A,B). Pre-treatment of HAECs with BPE did not significantly attenuate this effect (1.10 ± 0.08-fold; n = 3; *p* = 0.2) (Figure 3A,B). Neither Ang II or BPE treatment affected the expression of NOX2, NOX4, and NOX5 (Figure 3C–H).

### 3.5. BPE Increased the Expression of Antioxidant Enzymes in HAECs Treated with Ang II

**Figure 4:**
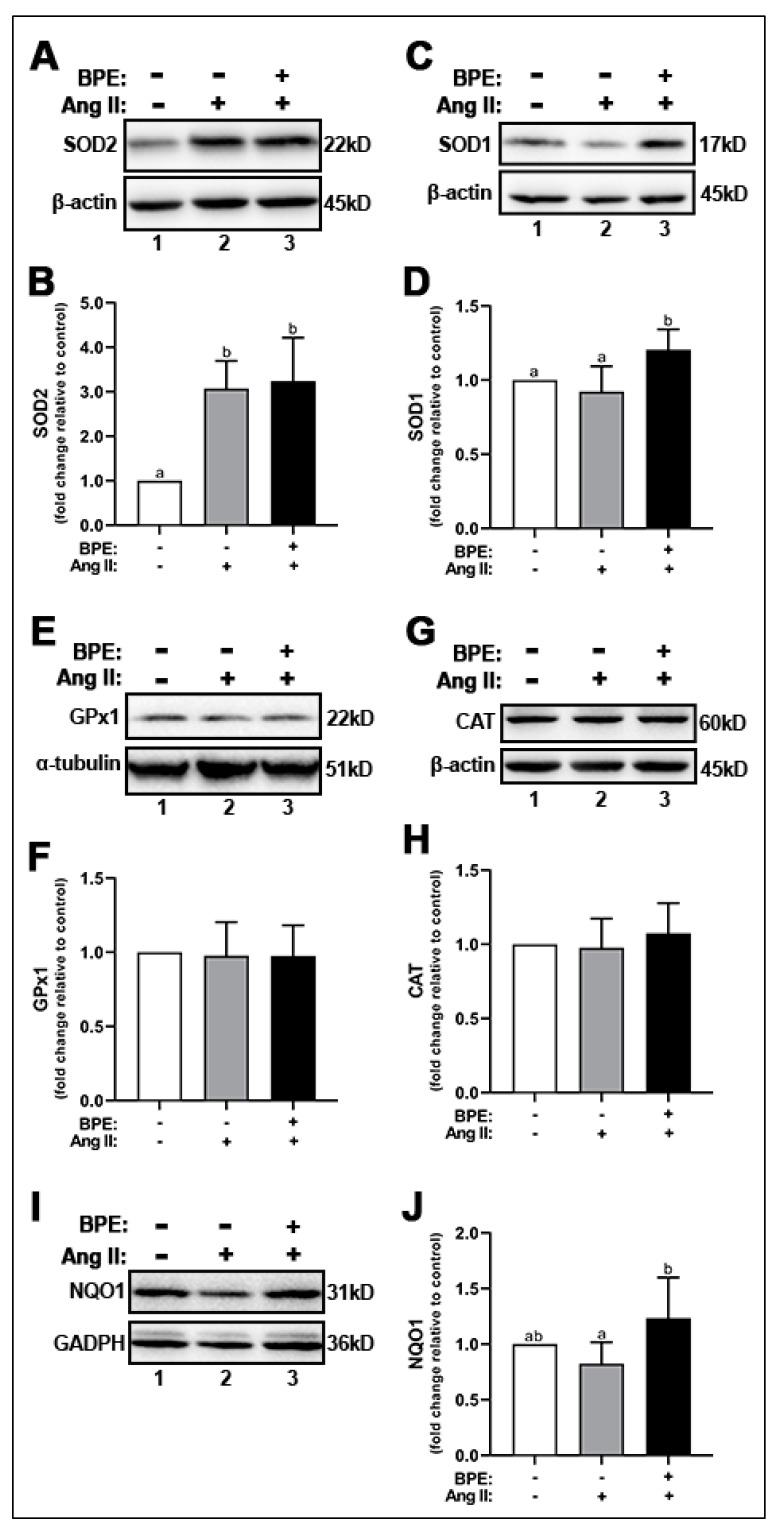
**Blueberry polyphenol extract (BPE) increases the expression of antioxidant enzymes in angiotensin (Ang) II-treated human aortic endothelial cells (HAECs).** HAECs were treated with 200 µg/mL of BPE for 1 h then treated with 200 nM of Ang II for 12 h. Protein expression of SOD2 (**A**,**B**), SOD1 (**C**,**D**), GPx1 (**E**,**F**), CAT (**G**,**H**), and NQO1 (**I**,**J**) were determined by Western blot. Quantification was performed using Image Lab (Bio-Rad Laboratories, Inc.). Data are expressed as mean ± SD from seven (SOD1, SOD2, and GPx1) four (CAT) and six (NQO1) independent experiments. Values that do not share the same letter are significantly different from each other (*p* ≤ 0.05).

Since BPE prevented the increase in O_2_^•−^ induced by Ang II but did not affect NOX expression, we hypothesized that BPE may increase the expression of antioxidant enzymes. Thus, we assessed the expression of the dismutases SOD1 and SOD2, peroxidases such as CAT and GPx1, and the cytosolic reductase NQO1. Ang II significantly increased SOD2 expression (3.07 ± 0.62-fold; n = 5; *p* = 0.001) in HAECs compared to control (Figure 4A,B). BPE did not elicit any further significant increases in Ang II-treated HAECs (3.24 ± 0.98-fold; n = 5; *p* = 0.9) (Figure 4A,B). BPE significantly increased the expression of SOD1 compared to Ang II-treated HAECs (1.21 ± 0.14 vs. 0.92 ± 0.17-fold, respectively; n = 6; *p* = 0.004) and control (*p* = 0.03) (Figure 4C,D). Expression levels of the peroxidases GPx1 (Figure 4E,F) and CAT (Figure 4G,H) were not affected by either Ang II or BPE. Ang II did not significantly affect the expression of NQO1 (Figure 4I,J). However, BPE elicited a significant increase in NQO1 expression in Ang II-treated HAECs (1.24 ± 0.36 vs. 0.82 ± 0.19-fold, respectively; n = 5; *p* = 0.04) (Figure 4I,J).

### 3.6. BPE Increased the Expression of NRF2 and Attenuated Ang II-Induced Increase in NF-κB Expression in HAECs

**Figure 5:**
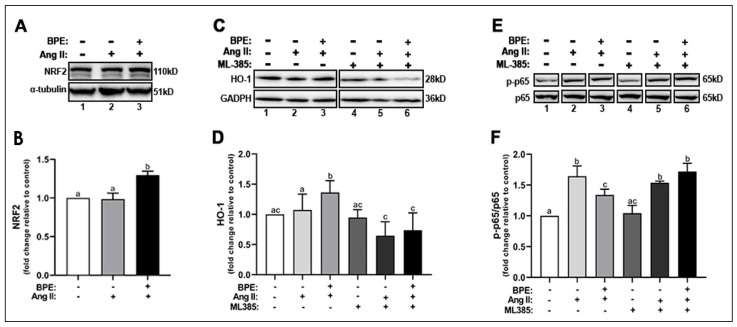
**Blueberry polyphenol extract (BPE) increases the expression of NRF2 and HO-1 while reducing NF-κB p65 phosphorylation in angiotensin (Ang) II-treated human aortic endothelial cells (HAECs).** HAECs were treated with 200 µg/mL of BPE for 1 h then treated with 200 nM of Ang II for 12 h. Protein expression of NRF2 (**A**,**B**), HO-1 (**C**,**D**), and NF-κB p65 (**E**,**F**) were determined by Western blot. Quantification was performed using Image Lab (Bio-Rad Laboratories, Inc.). Data are expressed as mean ± SD from nine (HO-1), and three (NRF2 and NF-κB) independent experiments. Values that do not share the same letter are significantly different from each other (*p* ≤ 0.05).

Next, we examine whether the increase in the expression of SOD1, SOD2, and NQO1 was in response to an increase in the activation of NRF2, the redox transcription factor that plays a role in transcribing genes for these antioxidant enzymes. Ang II did not change NRF2 expression compared to control while BPE significantly increased NRF2 expression compared to Ang II (1.30 ± 0.12 vs. 0.99 ± 0.17-fold, respectively; n = 5; *p* = 0.004) and control (*p* = 0.005) (Figure 5A,B). BPE also significantly increased the expression of HO-1, a cytoprotective enzyme transcribed by NRF2, compared to Ang II (1.36 ± 0.20 vs. 1.07 ± 0.26-fold, respectively; n = 11; *p* = 0.02) and control (*p* = 0.002) (Figure 5C,D). To confirm whether BPE is in fact exerting its beneficial effects on Ang II-treated HAECs through NRF2, we inhibited this transcription factor using the specific NRF2 inhibitor ML385 and measured HO-1 expression. ML385 did not elicit any changes in the absence of Ang II and BPE. However, inhibition of NRF2 by ML385 led to a significant decrease in HO-1 expression in the presence of Ang II alone (0.65 ± 0.23-fold; n = 7; *p* = 0.001) or with BPE (0.74 ± 0.29-fold; n = 7; *p* ˂ 0.0001) (Figure 5C,D).

Since increased oxidative stress can exacerbate the inflammatory response via activation of the redox transcription factor NF-κB, we measured the expression of the p-NF-κB p65 subunit. Ang II significantly increased the phosphorylation of the NF-κB p65 subunit (1.64 ± 0.17-fold; n = 3; *p* = 0.0001), which pre-treatment with BPE (1.34 ± 0.09-fold; n = 3; *p* = 0.02) was able to attenuate (Figure 5E,F). NRF2 inhibition did not affect NF-κB p65 subunit phosphorylation in the presence of Ang II but it further increased it in cells treated with Ang II and BPE (1.72 ± 0.13-fold; n = 3; *p* = 0.01) (Figure 5E,F). This may confirm that BPE exerts its effects in HAECS treated with Ang II in a NRF2-dependent mechanism.

## 4. Discussion

**Figure 6:**
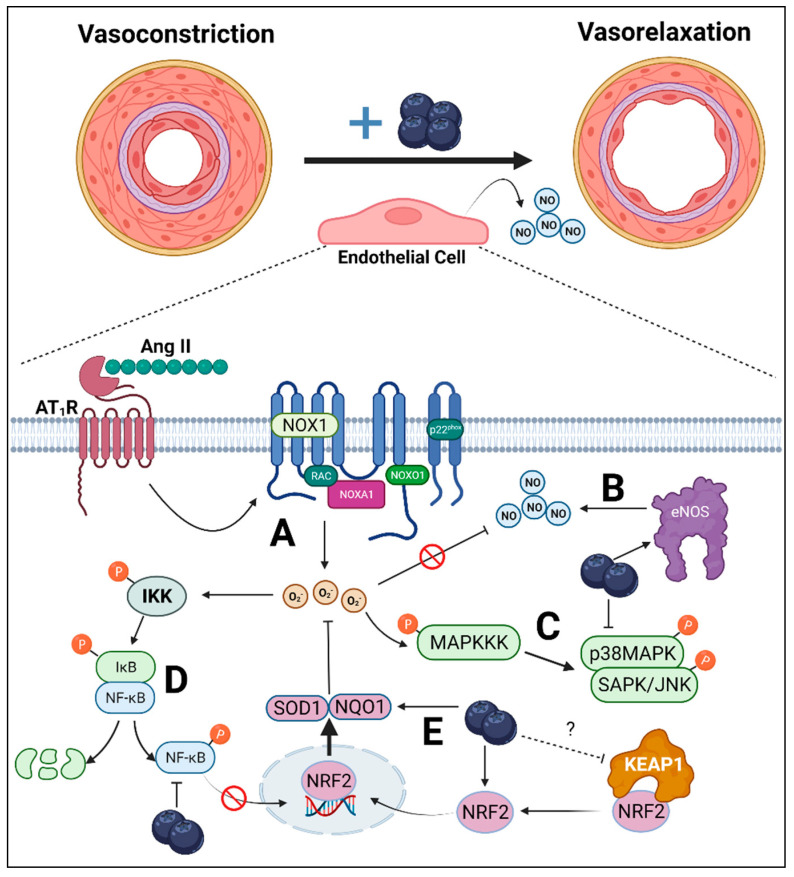
Overall endothelial-dependent mechanisms by which blueberry polyphenols may attenuate hypertension. Upregulated renin–angiotensin–aldosterone system results in the increased synthesis of angiotensin (Ang) II, which causes vasoconstriction due to reduced endothelial-derived nitric oxide (NO). (A) Ang II binds to Ang II type-1 receptor (AT_1_R), which results in the activation of NADPH-oxidase (NOX)1. NOX1 synthesizes superoxide anions (O_2_^•−^), which can react with NO, reducing NO bioavailability. (B) Blueberries may reverse this effect by increasing the synthesis of NO via increased endothelial NO synthase (eNOS) activity. (C) Mitogen-activated protein kinases (MAPKs), such as p38MAPK and stress-activated protein kinases (SAPK)/Jun amino-terminal kinases (JNK), are redox-sensitive kinases activated by upstream MAPK kinase kinase (MAPKKK). The phosphorylation of p38MAPK and SAPK/JNK expression was attenuated by blueberry polyphenols, suggesting reduced cellular oxidative stress. (D) Inhibitor of nuclear factor kappa B (IκB) kinase (IKK) is also a redox-sensitive kinase, which can undergo auto-phosphorylation upon interaction with reactive oxygen species (ROS) and is upstream of NF-κB. Following IKK phosphorylation, IκB is phosphorylated and the p65 subunit of NF-κB is then also phosphorylated. IκB then undergoes ubiquitin-dependent degradation, liberating NF-κB to translocate to the nucleus where it is involved in inflammatory transcription. Blueberries, however, reduced p65 phosphorylation, suggesting reduced IKK phosphorylation via reduced ROS. (E) These cytoprotective effects of blueberry polyphenols may be mediated by nuclear factor-erythroid factor 2-related factor 2 (NRF2), a master regulator of antioxidant enzymes. Kelch-like ECH-associated protein (KEAP)1 sequesters NRF2 in the cytosol and the entire complex undergoes ubiquitin-dependent degradation. However, conformational changes in KEAP1 prevent NRF2 binding, allowing NRF2 to translocate to the nucleus where it is involved in the transcription of antioxidant enzymes, NADPH dehydrogenase quinone (NQO)1 and superoxide dismutase (SOD)1, both of which can neutralize O_2_^•−^. Blueberries increase cellular NRF2 concentration possibly via interaction with KEAP1, resulting in increased NQO1 and SOD1, overall reducing NOX1-mediated oxidative stress preventing NO neutralization via O_2_^•−^.

Several human [26,27,39] and animal [30,31] studies have demonstrated the anti-hypertensive effects of blueberries. Thus, the main goal of this study was to investigate the mechanisms by which blueberry polyphenols exert their vasoactive effects using HAECs treated with Ang II. Our results, summarized in Figure 6, demonstrate that BPE attenuated the Ang II-induced decrease in NO metabolite levels, which was in part due to the increase in NOS activity. In addition, BPE prevented the increase in ROS induced by Ang II suggesting that BPE not only increases NO synthesis but also NO bioavailability. This decrease in ROS appears to be attributed to an increase in the expression of antioxidant enzymes including SOD1, NQO1, and HO-1, rather than a decrease in the expression of ROS production, as BPE did not significantly reduce Ang II-induced NOX1 expression. Further, our data suggest that the actions of BPE on the expression of antioxidant enzymes is in part mediated by NRF2. Lastly, we show that BPE attenuated ROS signaling as indicated by decreased activation of the redox MAPKs, SAPK/JNK and p38, and the redox pro-inflammatory transcription factor NF-κB, which was likely due to the reduction in ROS levels.

NO is the main endothelium-derived relaxing factor synthesized by eNOS. Ang II has been shown to decrease NO levels and Ser^1177^ p-eNOS expression in human umbilical vein cells (HUVECs) [40]. In the current study, we show that Ang II decreases NO levels, which was prevented by pre-treatment with BPE. To investigate whether these observations were due to Ang II and BPE effects on NO production, we measured NOS activity. Ang II had no effect on NOS activity while BPE significantly increased it. To our knowledge, no studies have reported on the effects of blueberry polyphenol extracts on NO levels and NOS activity in endothelial cells stimulated with Ang II. However, Park et al. [41] have shown that pterostilbene, a polyphenol abundant in blueberries, increased NO levels and Ser^1177^ p-eNOS expression in HUVECs. Since Ang II did not affect NOS activity in our study, we hypothesize that the decrease in NO levels was due to a decrease in NO bioavailability rather than its production. Therefore, we sought to measure ROS levels in our cells.

The increase in O_2_^•−^ induced by Ang II was significantly attenuated by pre-treatment with BPE. NADPH oxidases are a major source of ROS. NOX1, NOX2 and NOX5 are O_2_^•−^ generators while NOX4 produces primarily H_2_O_2_. Ang II has been shown to upregulate the expression of aortic NOX1 and NOX2. For example, deletion of NOX1 gene blunted Ang II-induced aortic O_2_^•−^ production and pressor response [42]. NOX2 knockout mice were also shown to have attenuated production of aortic O_2_^•−^ in response to Ang II compared to wild-type mice [43]. NOX4 appears to have a protective role in the vascular system as NOX4-deficient mice developed endothelial dysfunction [44]. In the present study, Ang II upregulated the expression of NOX1 but had no effect on the other isoforms. However, BPE was not able to fully attenuate this effect. We also observed a slight increase in NOX4 expression, albeit not significant. Further, we noted significant increases in H_2_O_2_ elicited by Ang II, which was not affected by BPE.

Because NOX expression was not reduced by BPE in the present study, we hypothesized that the attenuation in O_2_^•−^ levels was due to an increase in the expression of antioxidant enzymes such as SOD, which converts O_2_^•−^ to H_2_O_2_. Our data indicate that Ang II led to a significant increase in the expression of SOD2, a mitochondrial enzyme, which was not further augmented by BPE. However, Ang II had no effect on the expression of SOD1, a cytosolic enzyme, while BPE significantly increased it. Neither Ang II nor BPE affected the expression of peroxidases such as GPx1 and CAT, which may also explain the fact that H_2_O_2_ remained increased in the presence of BPE. We also show that BPE increased the expression of NQO1, a detoxifying enzyme that reduces quinones to hydroxyquinones. In addition to these enzymatic antioxidants mediated by BPE, the polyphenols may act as direct antioxidants. Indeed, blueberries have a relatively high FRAP score compared with many other fruits and vegetables [45]. In hypertensive women, blueberry consumption reduced 8-hydroxy-2′-deoxyguanosine (8-OHdG), a marker of oxidative stress-induced DNA damage, after four weeks [46]. In healthy males, consumption of blueberry postprandially reduced H_2_O_2_-induced DNA damage [47]. Nonetheless, the antioxidant effects from blueberries are likely synergistic with both enzymatic and non-enzymatic mechanisms. However, the enzymatic effects may be more important as evidenced by NRF2 genetic knockout studies with single bioactive compound supplementation [48,49].

Since the abovementioned antioxidant and detoxifying enzymes can be mediated by NRF2, we examined the effects of Ang II and BPE on its expression. We show that NRF2 expression is significantly increased by BPE. To confirm whether BPE is acting in a NRF2-dependent manner, we treated cells with the NRF2 inhibitor, ML385, and measured the expression of HO-1, a cytoprotective enzyme that protects against oxidative stressors. We show that BPE increased expression of HO-1, which was significantly suppressed by ML385 demonstrating the role of NRF2 in the beneficial actions of BPE. Ang II is known to upregulate redox-sensitive kinases and the NF-κB transcription factor. Since BPE was able to reduce O_2_^•−^ levels by increasing the expression of NRF2 and antioxidant enzymes, we hypothesized that BPE would decrease activation of MAPKs, and Akt as well as NF-κB. Our data indicate that BPE attenuated the Ang II-induced phosphorylation of SAPK/JNK and p38. Additionally, we show that BPE lessened the phosphorylation of the NF-κB p-65 subunit induced by Ang II, which was significantly increased with NRF2 inhibition.

NRF2 is continually synthesized by the cell, however, it is sequestered in the cytosol by KEAP1, which undergoes continuous ubiquitination and degradation when the KEAP1–NRF2 complex is formed. Under conditions of oxidative stress, modifications in cysteine residues on KEAP1 result in NRF2 accumulation in the cytosol and translocation to the nucleus [50]. As with NF-κB, NRF2 binds to cAMP response element binding protein (CREB)-binding protein (CBP) allowing it to bind to the transcriptional region of DNA encoding the ARE, leading to the transcription and synthesis of the aforementioned antioxidant enzymes [51]. Because CBP can bind to both NF-κB and NRF2, these proteins compete for binding [52]. However, under conditions of inflammation and oxidative stress, NF-κB appears to bind 10-fold greater to CBP than NRF2 as observed in HepG2 cells treated with 12-O-tetradecanoylphorbol-13-acetate [53]. Thus, under pathological conditions observed in hypertension, it can be assumed that NRF2 would be deprived of CBP binding, and under these circumstances, insufficient ARE transcription would occur, preventing NRF2 from eliciting an overwhelming enough antioxidant response to counteract ROS. Thus, activating NRF2 is of physiological importance.

The effects of blueberries on the expression of the proteins assessed in the current study in endothelial cells stimulated with Ang II has not been reported. However, blueberry polyphenols have been used in endothelial cell lines to reduce ROS under a variety of insults. For example, Sivasinprasasn et al. [54] showed that cyanidin-3-glucoside, a major anthocyanin in blueberries, reduced ROS induced by Ang II (1 µM) in a dose-dependent manner in immortalized umbilical endothelial cells (EA.hy926) [54]. This was accompanied by a dose-dependent increase in NRF2 and HO-1 expression an attenuation of Ang II-induced increase in the expression of the NF-κB p-65 subunit [54], which corroborates our findings. Myricetin, a blueberry flavonol, also reduced oxidative stress-induced by high-glucose conditions in human umbilical vein endothelial cells (HUVEC)s [55]. In addition, in HUVECs, NF-κB upregulation after stimulation with tumor necrosis factor (TNF)-α was reduced by both cyanidin-3-glucoside [56] as well as by malvidin and its glycosides [57]. Under basal conditions in HUVECs, blueberry anthocyanins, malvidin and its glycosides, reduced ROS production, increased total SOD, and increased HO-1 [58]. It is interesting to note that xanthine oxidase (XO), a ROS-producing enzyme, was also reduced. While we did not investigate the role of XO, it is possible that this was a contributing source of ROS in the present study. Landmesser et al. [59] demonstrated that XO is potentially redox-sensitive and can be activated by NOX, which may be of relevance in the present study. It was also demonstrated that Ang II stimulation (100 nM) in bovine aortic endothelial cells increased XO protein levels, which peaked at 12 h and coincided with increased superoxide production [59].

## 5. Conclusions

Collectively, our data indicate that blueberries preserve NO bioavailability and reduce Ang II-mediated oxidative stress and inflammatory signaling in HAECs. We also provide evidence that these protective effects may be mediated by increased NRF2 transcriptional activity. Thus, targeting NRF2 is likely of major clinical significance. For example, NRF2 agonist tert-butylhydroquinone (tBHQ) prevented hypertension and vascular oxidative stress in a two-week Ang II infusion model in mice [60]. However, NRF2 genetic knockout mice did not experience this protection following tBHQ, illustrating the importance of NRF2 activation in attenuating hypertension. We have demonstrated the anti-hypertensive effects of blueberry consumption clinically in a prior investigation [24]; however, the role of NRF2 in mediating these anti-hypertensive effects was not evaluated. As demonstrated in the present study in vitro with BPE, NRF2 and its activity is also increased in vivo following polyphenol-rich fruit consumption in peripheral blood mononuclear cells in human volunteers [61]. Anthocyanins, such as those found in blueberries, appear to be directly involved in NRF2-mediated ARE-activation [62], and this may be due to polyphenol–protein interaction with KEAP1, leading to NRF2 accumulation in the cell [63]. A number of clinical studies have been conducted utilizing NRF2 inducers, such as dimethyl fumarate, bardoxolone methyl, oltipraz, and sulforaphane [64]. However, blueberries are safe and free of side effects, comparatively low cost, and may be effective inducers of NRF2 activation clinically. Future studies should aim to investigate the NRF2-mediated role of blueberries in hypertension using genetically modified animal models as well as clinical studies.

## Figures and Tables

**Table 1:** Polyphenolic content and antioxidant capacity of blueberry polyphenol extract (BPE).

Total Polyphenol Content	Total Anthocyanin Content	Antioxidant Capacity (FRAP)
141.3 ± 3.4 µmol GAE/L	105.3 ± 2.0 C3GE mg/L	205.3 ± 4.7 µmol Fe^2+^/L

Data are expressed as mean ± SD from three independent experiments. GAE, gallic acid equivalents; C3GE, cyanidin-3-glucoside equivalents.

## Data Availability

Data is contained within the article.
